# The complete mitochondrial genome of *Gymnobelideus leadbeateri* (Mammalia: Petauridae)

**DOI:** 10.1080/23802359.2021.1875919

**Published:** 2021-02-12

**Authors:** Tao Wu, Yanpeng Chen, Sajeewa S. N. Maharachchikumbura

**Affiliations:** a Glasgow College, University of Electronic Science and Technology of China, Chengdu, P.R. China; b School of Life Science and Technology, University of Electronic Science and Technology of China, Chengdu, P.R. China

**Keywords:** Diprotodontia, *Gymnobelideus leadbeateri*, mitochondrial genome, phylogenetic analysis

## Abstract

The *Gymnobelideus leadbeateri* (Leadbeater’s Possum) is listed as Critical Endangered on the International Union for Conservation of Nature (IUCN) Red List. We assembled the complete mitochondrial genome for the *G. leadbeateri* and characterized it to provide informative data for forthcoming studies for understanding its evolution and conservation genetics. The *G. leadbeateri* mitogenome is 16,812 bp long and encodes 13 protein-coding genes (PCGs), two ribosomal RNA genes (12S rRNA and 16S rRNA) and 22 transfer RNA (tRNA) genes. Phylogenetic analysis based on complete mitogenome shows that *G. leadbeateri* is related to *Petaurus breviceps* (sugar glider) and *Dactylopsila trivirgata* (striped possum).

The *Gymnobelideus leadbeateri* McCoy is distributed only in Victoria state in southeast Australia (Harley 2004). Its population is estimated to be 1,100 ∼ 11,000 and the species mainly lives in old trees’ hollows (Woinarski and Burbidge 2016). *Gymnobelideus leadbeateri* has been listed on Critically Endangered IUCN Red List with a declining population across its range (Woinarski and Burbidge 2016), whereas its habitat is threatened by forest fire and timber industry (Lindenmayer et al. 1991). In present study, we assembled the mitochondrial genome of *G. leadbeateri* to know more about breeding and genetics of this species and protect it better.

Sample collected from a female Leadbeater’s possum at Yellingbo Nature Conservation Reserve, Australia (Latitude: 37°50′ S; Longitude: 145°29′ E) was provided by Monash University and sequenced by Deakin University (Accession no.: SAMN13475009). DNA extracted from liver, heart and muscle tissues was sequenced using NovaSeq 6000 sequencer. The DNA shield is stored at Monash University with accession B50252 and the body of this animal is stored at Healesville Sanctuary with the same accession in a freezer. The raw whole genome sequence data of *G. leadbeateri* was retrieved from GenBank (SRR10641223). The obtained reads were analyzed based on quality using FastQC v0.11.9 (Andrews 2010) and the filtered reads were assembled using GetOrganelle v1.7.1 (Jin et al. 2020). The search tool tRNAscan-SE v2.0.6 (Lowe and Chan 2016) and MFannot webserver (Beck and Lang 2010) (https://megasun.bch.umontreal.ca/cgi-bin/dev_mfa/mfannotInterface.pl) were used to annotate the mitogenome. All the genes were manually checked using online NCBI blastn tool.

The complete circular mitochondrial genome of *G. leadbeateri* is 16,812 bp in length with the GC content of 39.5%. The mitogenome consists of 13 protein-coding genes (PCGs), 2 ribosomal RNA genes (12S rRNA and 16S rRNA) and 22 tRNA genes. The proportions of A, T, G, and C are 33.7, 26.8, 12.4, and 27.1%, respectively. All PCGs use start codon (ATG, ATA and ATT) and 10 PCGs have the stop codon (TAA, TAG, AGA). The PCG order (*nad1*, *nad2*, *cox1*, *cox2*, *atp8*, *atp6*, *cox3*, *nad3*, *nad4L*, *nad4*, *nad5*, *nad6*, *cob*) is the same as two other species, *Dactylopsila trivirgata* and *Petaurus breviceps* (Munemasa et al. 2006).

For mitogenome phylogenetic analysis, ten species were chosen from three sister families of Diprotodontia: Petauridae, Macropodidae and Potoroidae. *Dromiciops gliroides* and *Procavia capensis* were used as the outgroup taxa. The mitogenome sequences were aligned using Mafft v7.471 (Katoh and Standley 2013) with the –auto option, and filtered using Gblocks v0.91b (Castresana 2000) with block options –b4 = 5, –b5 = h. Phylogenetic tree was inferred by IQ-TREE v1.6 (Nguyen et al. 2015) using maximum likelihood (ML) method with the best-fit model TIM2 + F + I + G4. The resulting tree was visualized by iTOL v4 (Letunic and Bork 2019). Phylogenetic analyses indicate that *G. leadbeateri* is closely related to *Petaurus breviceps* and *Dactylopsila trivirgata* (Figure 1). These data, which represent the first complete mitogenome for the *G. leadbeateri* provided in this study contributes to the phylogeny and protection of this Critical Endangered species.

**Figure 1.**
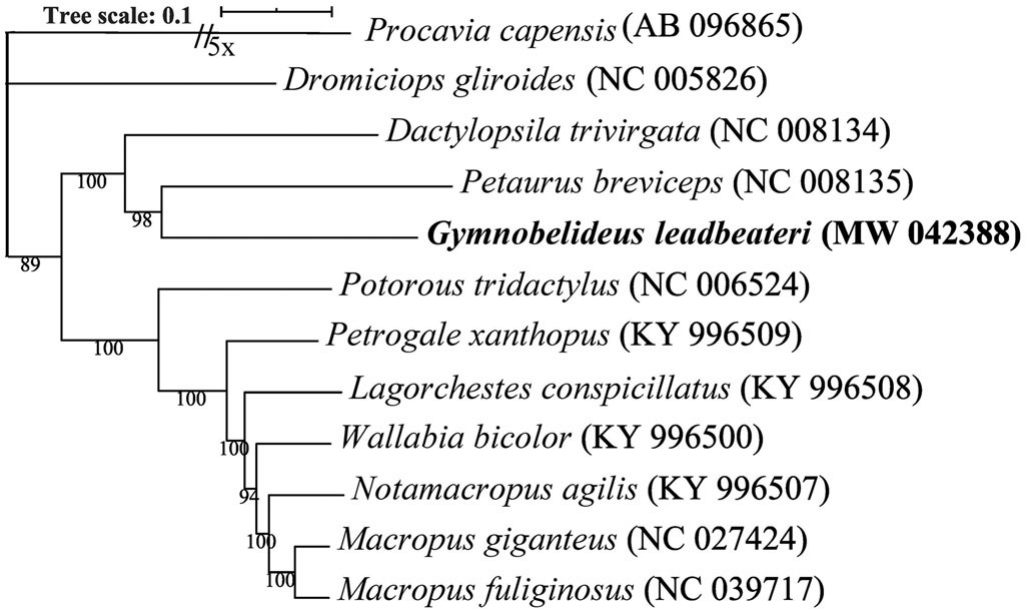
Phylogenetic tree inferred from the ML analysis of the complete mitochondrial genomes of the analyzed taxa belongs to the order Diprotodontia. The bootstrap support values are given at the nodes and the species name is followed by the strain GenBank accession numbers. Isolate from present study is in bold and the scale bar represents the expected number of changes per site.

## Data Availability

The data (annotated complete mitochondrial genome of *Gymnobelideus leadbeateri*) that support the findings of this study are submitted to NCBI GenBank (https://www.ncbi.nlm.nih.gov/) under accession number MW042388.
